# A Rare Cause of Chronic Rhinosinusitis Secondary to a Retained Pledget: A Case Report

**DOI:** 10.1155/crot/7411692

**Published:** 2025-08-14

**Authors:** Snehitha Talugula, Nicholas Callahan, Victoria S. Lee

**Affiliations:** ^1^College of Medicine, University of Illinois Chicago, Chicago, Illinois, USA; ^2^College of Dentistry, University of Illinois Chicago, Chicago, Illinois, USA; ^3^Department of Otolaryngology-Head and Neck Surgery, University of Illinois Chicago, Chicago, Illinois, USA

**Keywords:** chronic rhinosinusitis, inflammatory, rhinology

## Abstract

**Introduction:** Chronic rhinosinusitis (CRS) is a widely prevalent disease. Retained objects as the cause of CRS are not commonly reported in the literature. The impact of the removal of this object is also a point of interest. The purpose of this case report was to investigate a patient whose CRS was suspected to be due to a foreign object and his postoperative course.

**Case Presentation:** A 37-year-old male patient presented to the clinic with a chief complaint of right-sided nasal congestion, severe maxillary sinus pressure, and green thick nasal drainage for many years. He reported a history of dental and endonasal surgery on that side 8 years ago in Saudi Arabia. Imaging showed complete opacification of the right maxillary sinus and anteroinferior nasal cavity and several irregular-shaped radio-opaque materials within this region. His symptoms were refractory to medical management, and he chose to undergo endoscopic sinonasal surgery. A piece of gauze was removed from the right sinonasal cavity and maxillary sinus. In the weeks following surgery, his symptoms and inflammation fully resolved.

**Conclusions:** Retained objects or material within the nose and sinuses are not commonly reported in the literature. This patient's CRS was secondary to a preventable cause. His increased morbidity after his initial surgery 8 years ago could have been avoided with careful attention to surgical counts.

## 1. Introduction

The estimated prevalence of chronic rhinosinusitis (CRS) is between 2.1% and 13.8% in the United States, with roughly 7.3% suffering from severe CRS [1, 2]. For isolated maxillary sinusitis, 10%–40% of cases are attributable to an odontogenic cause [2]. Isolated chronic maxillary sinusitis due to a foreign object within the nasal sinuses, however, is not commonly reported in adults. A case series by Bär et al. [3] did demonstrate that displacement of dental implants into the nasal cavity and maxillary sinus can occur rarely, and patients often present with symptoms or radiographic evidence of sinusitis. Retained gauze or surgical sponges specifically are referred to as gossypibomas and have an incidence rate in the head and neck of roughly 4% [4]. One such case of a gossypiboma retained in the ethmoid sinus is presented by Gotwald et al. [5] in 2012. In this case, the lack of radio-opaque markings on the gauze prevented it from being recognized on imaging performed due to the patient's persistent sinonasal symptoms after sinus surgery. Schoendorf and Jungehuelsing [6] similarly presented a case where a mass in the left nasal cavity turned out to be a retained gauze from epistaxis control a few weeks prior. The gauze caused no discomfort or nasal obstruction in the patient and may have gone unnoticed for longer had it not been for the patient's uncontrolled epistaxis. Bhogaraju et al. [7] similarly presented a case of retained gauze within the canine fossa region present for 18 months following dental extraction with the only symptom being fullness in that region. Interestingly, these cases were without associated sinusitis. Based on the literature, when maxillary sinusitis due to a foreign object does occur, it is usually secondary to a dental procedure and the retained object is commonly a dental implant, grafting material, or partial or complete tooth, although this bias may be related to sample size [8]. In this report, we present a case of a patient with a retained gauze in his right sinonasal cavity and associated sinusitis in the context of previous dental and endonasal surgery.

## 2. Case Presentation

**Figure 1:**
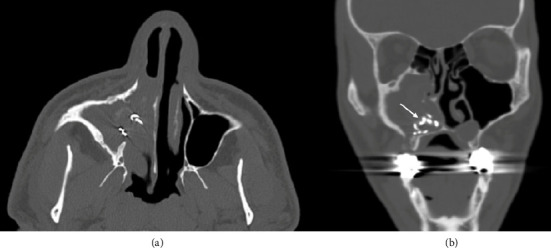
Axial (a) and coronal (b) noncontrast CT of the sinuses showing complete opacification of the right maxillary sinus and anteroinferior nasal cavity, irregular-shaped radio-opaque materials in the floor of the antrum consistent with retained foreign body (arrow), evidence of prior inferior meatal antrostomy to the maxillary sinus, and oroantral fistula.

A 37-year-old male patient presented to the clinic with a chief complaint of right-sided nasal congestion, severe maxillary sinus pressure, and green thick nasal drainage for many years. He reported a history of dental and endonasal surgery on the ipsilateral side 8 years ago in Saudi Arabia but does not recall any further details including the indication for surgery. He had no other past medical history, denied taking any medications, and was otherwise healthy. CBC with differential, lipid panel, and comprehensive metabolic panel were all within normal limits. Nasal endoscopy completed in the clinic was significant for severe edema of the anteroinferior nasal cavity; superiorly, the middle meatus showed moderate edema, and no obvious foreign object was seen in the sinonasal cavity. A CT scan was obtained which showed complete opacification of the right maxillary sinus and anteroinferior nasal cavity, irregular-shaped radio-opaque materials in the floor of the maxillary sinus and nose consistent with a retained foreign body, evidence of prior inferior meatal antrostomy into the maxillary sinus, and oroantral fistula (Figure 1). The metallic parts were on average 613 Hounsfield units (HUs) and the nonmetallic parts were on average 53 HUs. Our neuroradiologist, however, stated that the metallic parts elicit significant artifacts that render the HU measurements inaccurate/unreliable. Cultures were obtained at the time of surgery. Aerobic culture was positive for moderate *Eikenella corrodens* and rare *Corynebacterium striatum*; anaerobic culture showed many *Prevotella oris*. These bacteria are found in the oral cavity, and their presence within the sinonasal cavity was likely reflective of the odontogenic source of the patient's initial sinusitis precipitating the unspecified dental and endonasal surgery, rather than new microbes associated with the retained pledget. Initial treatment consisted of oral antibiotics (levofloxacin 500 mg orally daily for 2 weeks) and oral steroids (methylprednisolone 9-day taper starting at 24 mg for 3 days followed by 16 mg and then 8 mg each for an additional 3 days). Nasal steroid sprays and saline irrigations twice daily were also prescribed. There was no significant improvement in symptoms or on endoscopy with medical treatment, and the patient proceeded with surgery.

**Figure 2:**
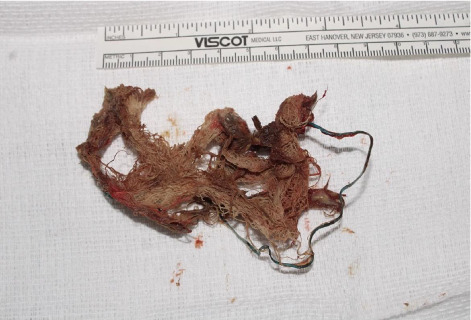
Retained gauze.

**Figure 3:**
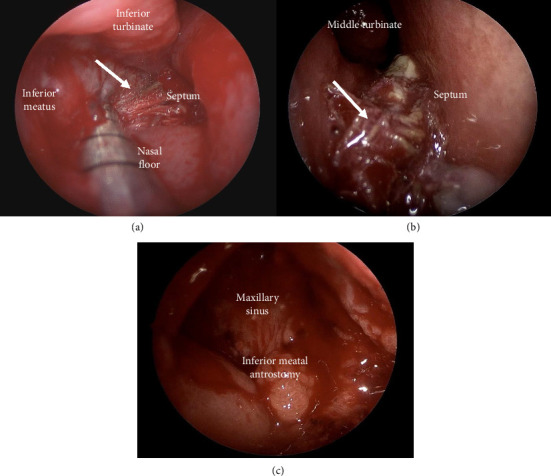
Endoscopic images of the right sinonasal cavity before (a), during (b), and after (c) removal of gauze (arrow).

The patient was taken to the operating room by the otolaryngology and oral maxillofacial surgery services. A foul-smelling piece of gauze was removed from the right sinonasal cavity and maxillary sinus (Figure 2). Endoscopic views show the gauze before (Figure 3(a)), during (Figure 3(b)), and after (Figure 3(c)) removal, with the prior inferior meatal antrostomy into the maxillary sinus now visible. Intraoperatively, visualizing through the inferior meatal antrostomy, severe inflammation and purulence of the maxillary sinus diffusely were noted. Thus, a middle meatal antrostomy into the maxillary sinus was performed, requiring a concha bullosa resection for middle meatal access, ensuring a widely patent drainage pathway for the maxillary sinus incorporating the natural os. Patency of the nasolacrimal system via probing and irrigation was confirmed given the presence of the previous inferior meatal antrostomy, with clearing of postobstructive secretions, presumably due to obstruction of Hasner's valve related to the mass effect of the foreign body and associated inflammation. Afterward, the patient was turned over to oral maxillofacial surgery for closure of the 2–3 mm in diameter oroantral fistula via standard two-layer closure with involution of the fistula, placement of a Gelfoam plug, and buccal advancement flap. The patient had complete resolution of his symptoms and inflammation on endoscopy postoperatively. The patient is followed yearly—most recently in March 2025—and remains asymptomatic without disease on endoscopy.

## 3. Discussion

A patient with persistent sinonasal symptoms of greater than 12 weeks with inflammation noted on imaging or endoscopy can be diagnosed with CRS [1]. Our patient had longstanding symptoms refractory to medical management with CT and endoscopy findings showing a retained foreign body, in this case, a gauze, and inflammatory changes within his right maxillary sinus. Sixty percent of foreign bodies in the maxillary sinus are secondary to dental treatments of some sort including tooth roots, dental implants, and other dental materials [8]. For these patients, a review by Hara et al. [8] found that 57.2% of patients present in under 1 month with symptoms related to the retained object, while only 7.5% of patients go over 5 years without discovery. In this case, both the duration of symptoms/presence of the foreign body and that the foreign body was a retained gauze are unusual. Our patient presented after 8 years, and it is unclear what prevented him from seeking treatment sooner.

The removal of the foreign body is performed surgically and is most commonly performed either by functional endoscopic sinus surgery (FESS) or by the Caldwell–Luc procedure, where an incision is made above the teeth to enter the maxillary sinus to retrieve the object. Both are described to have similar efficacy in the literature, and the decision of which to pursue includes factors such as foreign body location and surgeon preference. Caldwell–Luc procedures carry increased rates of postoperative sinusitis and facial swelling/numbness and are less frequently used with the advancement of minimally invasive endoscopic technology and procedures [9]. Furthermore, FESS may hold less risk in comparison to the Caldwell–Luc procedure as evidenced in a 10-year retrospective analysis of patients who underwent FESS for retrieval of displaced dental implants; in this study, no one developed postoperative complications at follow-up periods of 3 months–8 years [10]. FESS was chosen in this case as the location of the gauze was easily accessible and removed with this more minimally invasive approach.

Gossypibomas are a preventable complication. A variety of factors influence the epidemic of retained gauze postoperatively. For instance, breakdown in communication in the operative room leading to improper counts is a systems issue that can result in impaired patient safety [4]. Furthermore, when soaked with blood, the sponge or gauze itself can be indistinguishable from surrounding structures and thus easily missed by the surgeon. The latter problem is preventable with the use of radiofrequency chips on the pads themselves, which can be detected in X-rays obtained in the event of initially unreconciled sponge counts. A systematic review found that implementation of radiofrequency chips decreased the occurrence of unreconciled sponge counts by 71.3%, not only reducing time searching for missing gauze but also decreasing the likelihood of sponges being left in patients [11]. Imaging of our patient did detect radio-opaque tagging indicative of a radiofrequency chip. Systemic issues, such as intraoperative team communication, inaccurate sponge counts, lack of X-ray, and other measures in the event of unreconciled sponge counts, may have contributed. On his presentation to our clinic, the presence of the radiofrequency chip was indicative of the fact that his refractory sinusitis was secondary to a foreign body rather than an autologous process, as the HUs in this patient's CT scan were highly variable, given the significant artifact from the metallic parts of the gauze.

To our knowledge, the retained gauze from prior sinus surgery is a rare finding and is not described in depth within the current literature. This case underscores (1) the importance of careful examination of surgical sites prior to completion to prevent morbidity and infection that can occur from retained foreign objects and (2) the importance of accurate sponge counts and protocols in the event of missing sponges, such as X-ray, to minimize these occurrences.

## 4. Conclusion

In this report, we present the case of a patient who suffered from right-sided nasal congestion, severe maxillary sinus pressure, and green thick nasal drainage for 8 years since undergoing dental and endonasal surgery. The cause of his symptoms was found to be due to a retained gauze. After removal, his symptoms and inflammation resolved. This case highlights the importance of careful examination of surgical sites and proper counts of sponges and other foreign bodies during and especially at the end of an operation.

## Data Availability

Data sharing is not applicable to this article as no datasets were generated or analyzed during the current study.
